# COVID-19 in an adult with right isomerism and Fontan circulation: Successful management using risk stratification

**DOI:** 10.20407/fmj.2025-019

**Published:** 2025-11-05

**Authors:** Kazuyoshi Saito, Sayuri Yamabe, Arisa Kojima, Hidetoshi Uchida, Yuki Takigawa, Chihiro Nakashima, Meiko Hoshino, Kayoko Takada, Shigefumi Fukui, Tetsushi Yoshikawa, Hideo Izawa, Akira Yamada

**Affiliations:** 1 Department of Pediatrics, School of Medicine, Fujita Health University, Toyoake, Aichi, Japan; 2 Department of Cardiology, School of Medicine, Fujita Health University, Toyoake, Aichi, Japan

**Keywords:** COVID-19, Adult congenital heart disease, Fontan, Right isomerism, Risk stratification

## Abstract

Reports regarding coronavirus disease 2019 (COVID-19) with Fontan circulation are limited. Most studies indicate a relatively good clinical outcome in which SARS-CoV-2 infection is abated; however, COVID-19 can still cause severe pneumonia, and some studies report circulatory breakdown associated with acute respiratory distress syndrome in patients with Fontan circulation.

We present the case of a 32-year-old Japanese woman with right isomerism and a single right ventricle who had undergone a fenestrated extracardiac total cavopulmonary connection (Fontan operation), atrioventricular valve replacement, and pacemaker implantation. Despite receiving three doses of the severe acute respiratory syndrome coronavirus 2 mRNA vaccine, the patient contracted COVID-19 and presented with pneumonia. Although her symptoms were mild, the patient was classified as high-risk based on the European Society of Cardiology risk stratification guidelines for COVID-19 in patients with adult congenital heart disease and was admitted to the hospital. Initially, the patient received only symptomatic treatment. However, 2 days later, the patient’s COVID-19 condition worsened to moderate grade level II (SpO_2_ ≤93%, oxygen demand), with SpO_2_ dropping from 95% to 88% on room air. Treatment with remdesivir and dexamethasone following Japanese treatment protocols resulted in clinical improvement and discharge without hemodynamic complications.

COVID-19 pneumonia risks disrupting Fontan circulation in affected patients. This case illustrates the importance of prompt risk stratification using anatomical and physical evaluation and adherence to treatment guidelines in the management of patients with Fontan circulation and COVID-19.

## Introduction

An international registry study showed that the mortality rate of coronavirus disease 2019 (COVID-19) in patients with adult congenital heart disease (ACHD) is comparable to that of the general population.^1^ Despite the lack of conclusive evidence that Fontan circulation is a risk factor for severe COVID-19, the condition—characterized by the absence of a pulmonary ventricle—may increase susceptibility to hemodynamic disruption. Although many patients with COVID-19 and ACHD have shown favorable outcomes, instances of acute respiratory distress syndrome (ARDS) and hemodynamic disruption have also been reported.^2,3^ The risk of severe COVID-19 has since declined, but the infection can still cause severe pneumonia.

An international registry of 1,044 patients with COVID-19 and ACHD in Europe and the United States that was collected before the end of 2020 comprised 118 patients with Fontan circulation. The average patient age was 29 +/– 7.7 years old; the COVID-19 mortality rate was 3%, with an additional 6% who experienced severe illness.^1^

Right isomerism is a congenital condition in which organs on both sides of the body’s left–right axis display features that are usually associated with normal right-sided organs; typically, the spleen is not present (asplenia syndrome). Because the spleen is an important immune organ, these patients are immunocompromised and are reported to have a 1.62 times higher risk of mortality with COVID-19 compared with the other patients without right isomerism.^4^

Here, we report a case of COVID-19 in a postoperative patient with Fontan circulation and right isomerism, including asplenia syndrome. Although the patient developed pneumonia, adherence to the risk stratification and treatment protocols of the European Society of Cardiology (ESC) position paper for COVID-19 and the Japanese “Guide to the Treatment of Novel Coronavirus Infections (9th Edition)” allowed patient recovery without progression to severe disease.

## Case Presentation

### Admission related to COVID-19

The patient was a 32-year-old Japanese woman who had undergone a Fontan operation when she was 8 years old.

The patient’s condition and medical history were characterized by the following: situs ambiguous, L-loop, L-position; single atrium; unbalanced atrioventricular septal defect (functionally single right ventricle); double outlet right ventricle; pulmonary stenosis; right aortic arch; bilateral supra vena cava; post-operative left supra vena cava (LSVC) occlusion; common atrioventricular valve (CAVV) replacement (On-X^®^); and pacemaker implantation (DDD mode) for advanced atrioventricular block.

The patient’s surgical history that addressed these conditions was as follows. At one year of age, the patient underwent left Blalock–Taussig shunt surgery; at 7 years of age, she underwent a bilateral bidirectional Glenn shunt operation; and at 8 years of age, she underwent a fenestrated total cavopulmonary connection (22 mm Hemashield conduit^®^). LSVC obstruction was noticed soon after total cavopulmonary connection. At 11 years of age, the patient underwent plastic surgery for steel plate correction of scoliosis. For many subsequent years, the patient’s postoperative course was uneventful, and she was monitored only on an outpatient basis. The patient exhibited normal neural development; she worked full-time and lived independently as an adult.

However, at 31 years of age, the patient developed severe CAVV regurgitation that necessitated artificial valve replacement and an advanced atrioventricular block that caused intermittent syncope. The following preoperative catheterization findings were observed: central venous pressure, 13 mmHg; mean pulmonary arterial pressure, 13 mmHg; right ventricle pressure, 112/10 mmHg; ascending aorta, 112/60 (82) mmHg; pull-back pressure gradient, 0 mmHg (between pulmonary artery [Glenn] shunt or pulmonary artery conduit); Right ventricular ejection fraction, 53%; pulmonary artery resistance, 2.0 WU·m^2^; pulmonary blood flow/systemic blood flow ratio, 0.9; cardiac index, 1.6 L/m^2^/min; and CAVV regurgitation, grade III.

During surgery, artificial valve replacement (On-X^®^) was performed, and an epicardial pacemaker (DDD mode) was implanted. The operation, which was performed 5 months before the patient contracted COVID-19, was successful, and the postoperative course was uneventful.

The patient’s daily medications for heart failure included furosemide, spironolactone, aspirin, warfarin, and bisoprolol, and her hemodynamics were stable after AVV replacement and pacemaker implantation.

In July 2022, the patient presented with fever, cough, and sore throat as main symptoms. The patient had been in close contact with persons with COVID-19 2 days prior, during Japan’s fifth wave of the pandemic that predominantly featured the Omicron BA5 strain. The patient had already been vaccinated with the coronavirus mRNA vaccine three times; however, the vaccines were not as effective in preventing severe disease caused by the Omicron BA5 strain as it was with the pre-Omicron strains. On the day of hospitalization (Day 1), the patient had a fever of 38°C in the morning. Her family doctor prescribed antipyretics and antibiotics, which temporarily relieved the fever. However, given that the patient’s fever had risen to 39°C and was accompanied by cough, sore throat, chills, and fatigue by evening, the patient was taken to our emergency room by ambulance. At that time, the patient did not exhibit dyspnea.

Upon admission, the findings from physical examination of the patient were almost normal except for high fever and slightly wet extremities, as follows: height, 153 cm; weight, 48 kg (body mass index=20.5); body temperature, 38.4°C; heart rate, 101 bpm; blood pressure, 94/47 mmHg; respiratory rate, 17 breaths/min; SpO_2_, 95% (room air); intact consciousness; clear lung sounds; mechanical heart sounds with no gallop; no hepatomegaly; although no cold sensation and no edema in the extremities, slightly wet; no conjunctival jaundice; no anemia; mild pharyngeal redness; no lymphadenopathy; mild cervical vein dilation (not worsened); and a capillary refill time of <2 s.

**Figure 1:**
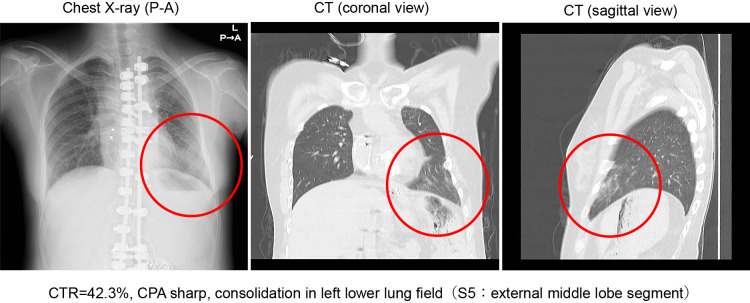
Chest X-ray and chest computed tomography imaging on admission Chest X-ray (posterior–anterior view) and chest computed tomography (coronal and sagittal views) reveal a consolidation in the left lower lung field (S5, external middle lobe segment). The cardiothoracic ratio (CTR) is 42.3%. CPA, costophrenic angle

A rapid antigen test was positive for COVID-19, and laboratory blood analysis results were as follows: white blood cell count, 14,100/ml; neutrophils, 96.6%; C reactive protein, 1.79 mg/dL; prothrombin time/international normalized ratio, 2.14; D-dimer, 2.3 μg/mL; and N-terminal pro-brain natriuretic peptide, 338 pg/mL (Table 1). Furthermore, SpO_2_ was 95% in room air (equivalent to baseline), and no CO_2_ retention was observed (pH, 7.419; pCO_2_, 40.4 mmHg; and HCO_3_^–^, 26.1 mmol/L in venous blood gas analysis), indicating no significant respiratory failure. Venous blood gas assay showed only mild lactic acid elevation (Lac, 2.34 mmol/L). A chest X-ray and computed tomography scan revealed decreased permeability in the left inferior lung field, indicating pneumonia in the left lateral middle lobe (S5; Figure 1).

**Figure 2:**
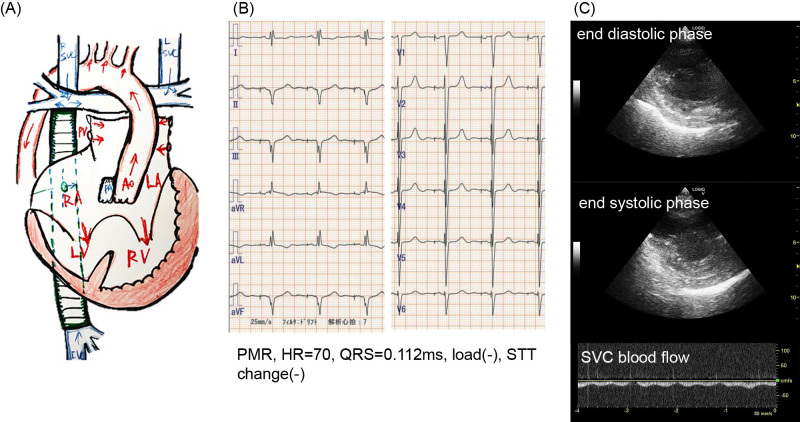
Cardiac assessment (A) A schematic diagram that illustrates the patient’s heart (S/P TCPC, Fontan operation). (B) 12-lead ECG on admission. (C) UCG revealed good ventricular contraction, effective opening and closing of mechanical valves, no pericardial effusion, and no retrograde blood flow in the SVC and IVC. Given the constraints of the COVID-19 isolation ward, a standard cardiology ultrasound system was not utilized. TCPC, total cavopulmonary connection; PMR, pacemaker rhythm; SVC, supra vena cava; IVC, inferior vena cava; S/P, status post; ECG, electrocardiogram; UCG, ultrasound cardiography.

An electrocardiogram showed a heart rate of 70 bpm in a pacemaker rhythm with no ST-T changes. Echocardiography demonstrated good ventricular contraction, proper mechanical valve function, no retrograde blood flow in the superior or inferior vena cava, and no pericardial effusion, indicating adequate ventricular function (Figure 2).

Based on the above and knowing the patient’s medical history regarding right isomerism and Fontan circulation the following diagnosis was made: right isomerism status post (S/P) Fontan operation, S/P common atrioventricular valve replacement, S/P epicardial pacemaker implantation (DDD mode), and acute pneumonia caused by COVID-19 (localized to the left lateral middle lobe, S5) that was classified as moderate (grade I) COVID-19.

### Treatment and clinical course of COVID-19

As described above, the patient’s COVID-19 symptoms appeared relatively mild on Day 1, and her daily medication was continued. However, as a high-risk patient as defined by the ESC position paper (Supplementary Figures 1 and 2), the patient was admitted to a dedicated COVID-19 ward where intravenous access was established, antitussive and expectorant medications were administered, and her condition was closely monitored. By Day 2, the patient’s fever decreased, and symptoms such as cough became milder; however, SpO_2_ declined to 88%–91% in room air in the evening. Nasal oxygen administered at 2 L/min improved SpO_2_ to 93%–95%. On Day 3, SpO_2_ decreased to 88% with the application of nasal oxygen of 2L/min, and the patient’s cough persisted; however, her subjective symptoms did not otherwise worsen. Based on consultation with the Department of Infectious Disease and ACHD specialists, consideration of the risk of acute lung injury or disruption of Fontan circulation, and Japan’s Ministry of Health, Labour and Welfare medical practice guide for COVID-19 that classified the patient’s COVID-19 as moderate (level II), remdesivir (200 mg on the first day and 100 mg/day thereafter) and dexamethasone (6 mg/day intravenously for 5 days) were administered (https://www.mhlw.go.jp/content/001248424.pdf ). By Day 4, the patient’s fever had resolved, and most other symptoms showed significant improvement. From Day 6, SpO_2_ normalized, and drug administration was completed by Day 7. On Day 8, the patient’s COVID-19 symptoms had not returned, and she was discharged. No long-term complications have been observed over the subsequent 3 years.

## Discussion

We report a case of COVID-19 in a patient with ACHD with right isomerism after a Fontan operation. To the best of our knowledge, no previous case reports have described a similar situation.

The treatment strategy was determined using risk stratification based on the ESC position paper for managing COVID-19 in patients with ACHD and the “Guide to the Treatment of Novel Coronavirus Infections (9th Edition)” in Japan.^5,6^ According to the ESC position paper, patients with congenital heart disease are evaluated based on the structure (anatomy, A) and function (physiology, P) of the heart (Supplementary Figures 1 and 2). Patients with a single ventricle are classified as “structurally high risk,” and those with Fontan circulation are classified as “functionally high risk” given the potential for chronic right heart failure.^6^ For high-risk patients with ACHD and COVID-19, hospitalization is recommended even in the absence of symptoms, and an early treatment plan should be made in consultation with an ACHD specialist. In this case, after consulting an ACHD specialist, treatment was guided by existing reports and the general guidance in Japan for treating patients with COVID-19.^5,6^ The selected treatment, which proved effective, included hospitalization and supportive care despite mild illness, followed by the administration of remdesivir and dexamethasone at the earliest signs of disease progression.

Under the current Japanese vaccination system, coronavirus infection is classified as a Category B disease, similar to seasonal influenza infection. Therefore, people over 65 years of age or those over 60 years with underlying diseases can receive public subsidies, if requested. Other patients, including the patient presented here, are responsible for the full cost of SARS-CoV-2 vaccination. The American Heart Association, the Centers for Disease Control and Prevention in the United States, the European Society of Cardiology and the International Society for Adult Congenital Heart Disease in Europe, and the Japan Society of Pediatric Cardiology recommend the coronavirus vaccine for maintaining pulmonary blood flow, preventing blood clots, and stabilizing oxygenation after Fontan surgery in patients with COVID-19. Therefore, we plan to recommend yearly vaccination for this patient along with thorough counseling on its benefits and risks.

In summary, this case underscores that, when appropriate precautions are taken and established protocols are followed, adult patients with right isomerism and Fontan circulation can recover from moderate (level II) COVID-19 without progression to severe disease or long-term sequelae.

## Figures and Tables

**Table 1:** Laboratory findings at admission

Peripheral blood test		Blood biochemical test			
WBC	14,500/μL	TP	7.2 g/dL	Glu	131 mg/dL
Eo	0.2%	Alb	4.3 g/dL	TG	70 mg/dL
Neut	96.6%	AST	40 IU/L	T-Chol	160 mg/dL
lympho	2.8%	ALT	28 IU/L	HDL	69 mg/dL
Mono	0.1%	LDH	405 IU/L	LDL	73 mg/dL
RBC	4.79×10^6^/μL	CK	73 IU/L	TSH	2.921 μIU/ml
Hb	15.3 g/dL	ChE	260 IU/L	fT3	2.91 pg/ml
Ht	45.5%	BUN	13.3 mg/dL	fT4	1.31 μg/dL
Plt	16.5×10^4^/μL	Cr	0.75 mg/dL	TIBC	1.7 μg/dL
Coagulation fibrinolysis test		Na	139 mEq/L	UIBC	0.5 μg/dL
PT	35%	K	3.2 mEq/L	Fer	41 mg/dL
PT-INR	2.14	Cl	103 mEq/L	Venous blood gas analysis	
APTT	33.2 seconds	Ca	9 mg/dL	pH	7.419
Fibrinogen	310 g/dL	P	2.9 mg/dL	pCO_2_	40.4 mmHg
D-dimer	2.3 μg/mL	Fe	128 mg/dL	HCO_3_^–^	26.1 mEq/L
Culture		CRP	1.79 mg/dL	BE	1.4 mEq/L
Blood culture	(–)	NT-proBNP	338 pg/mL	Lactate	2.34 mmol/dL
Urine culture	(–)	HANP	78.6 pg/mL	O_2_ sat	61.7%

WBC, white blood cell; Eo, Eosionophil; Neut, neutrophil; Lympo, lymphocyte; Mono, monocyte; RBC, red blood cell; Hb, hemoglobin; Ht, hematocrit; Plt, platelet; PT, Prothrombin Time; PT-INR, Prothrombin Time - International Normalized Ratio; APTT, Activated Partial Thromboplastin Time; TP, total protein; Alb, albumin; AST, Aspartate Aminotransferase; ALT, Alanine Aminotransferase; LDH, Lactate Dehydrogenase; CK, Creatine Kinase; ChE, Cholinesterase; BUN, Blood Urea Nitrogen; Cr Creatinine; Na, Sodium; K, Potassium; Cl, Chloride; Ca, Calcium; P, Phosphorus; Fe, Iron; CRP, C-Reactive Protein; NT-proBNP, N-terminal pro B-type Natriuretic Peptide; HANP, Human Atrial Natriuretic Peptide; Glu, Glucose; TG, Triglyceride; T-Chol, Total Cholesterol; HDL, High-Density Lipoprotein; LDL, Low-Density Lipoprotein; TSH, Thyroid Stimulating Hormone; fT3, Free Triiodothyronine; fT4, Free Thyroxine; TIBC, Total Iron-Binding Capacity; UIBC, Unsaturated Iron-Binding Capacity; Fer, Ferritin; pCO_2_, Partial Pressure of Carbon Dioxide; HCO_3_^–^, Bicarbonate; BE, Base Excess; O_2_ sat, Oxygen Saturation
