# Supplementary material for: COVID-19 in an adult with right isomerism and Fontan circulation: Successful management using risk stratification
Source: Fujita Med J. 2025 Nov 5;12(1):79–83. doi: 10.20407/fmj.2025-019 (PMC12865284; doi:10.20407/fmj.2025-019)
Supplement: Supplementary file 1 — Supplementary Materials [file fmj-12-079-s001.pdf]

S-Figure1 (© Eur Heart J 2021; 42: 1858-65. with permission to reuse) [6]

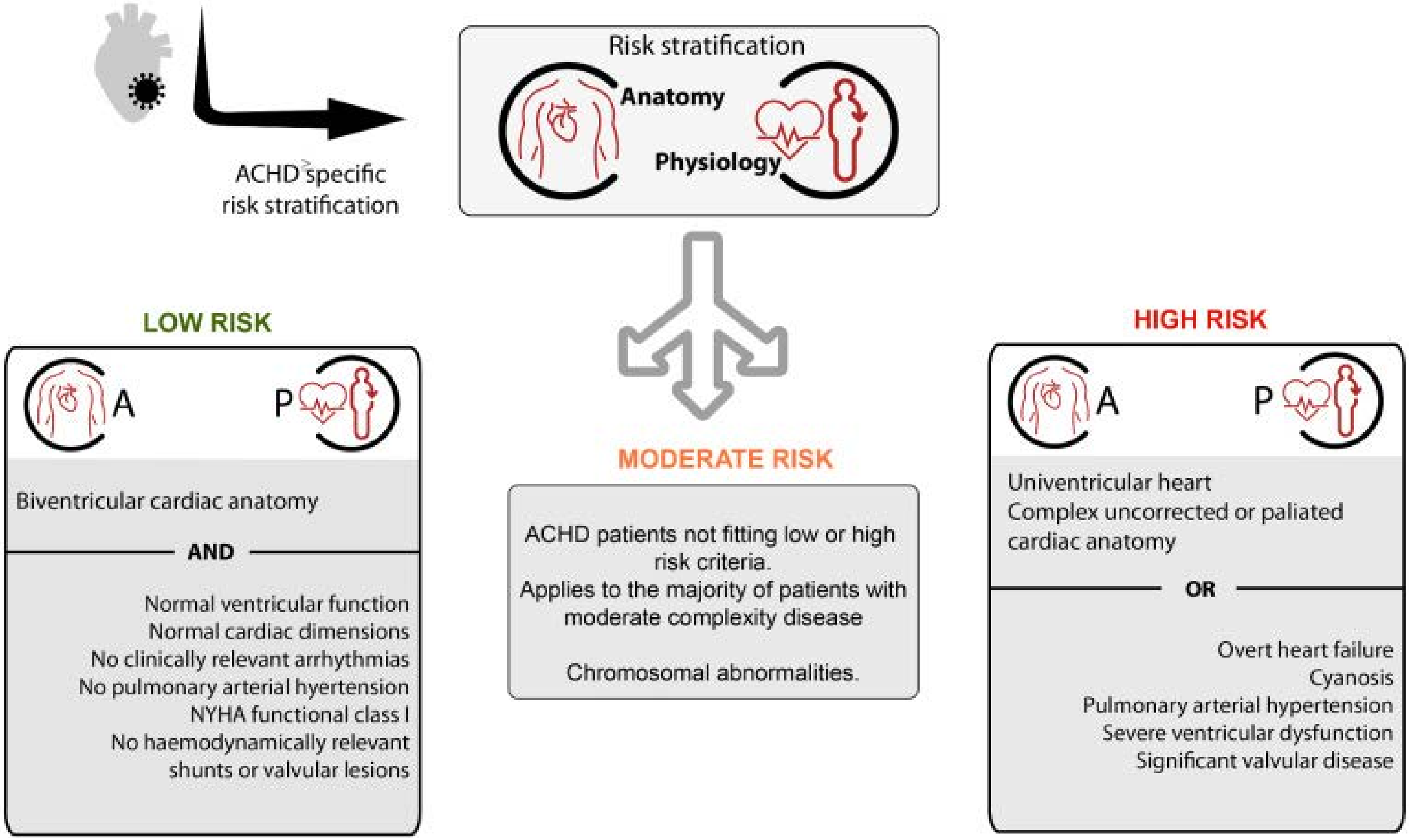

Supplementary Figure 1. Risk stratification of COVID-19 in patients with ACHD based on the position paper by the European Society of Cardiology [6]

Risk stratification is performed according to the evaluation of patients' anatomy (A) and physiology (P). The figure is included in Reference 6.

S-Figure2 (© Eur Heart J 2021; 42: 1858-65. with permission to reuse) [6]

Table 1 Recommendations for work/education and general medical management according to patient risk category

General considerations regarding work/education	Therapy in case of SARS-CoV-2 infection:
**Low-risk ACHD patients**	
Usual protection measures recommended	If clinically stable offer remote home management
Follow general recommendations (face mask, etc.)	Contact with ACHD centre advisable
No general limitation for work/school	Early admission in case of clinical deterioration
**Moderate-risk ACHD patients**	
Individualized risk assessment. General recommendations:	Consider early admission (even if oligosymptomatic)
- Reduce non-essential contact with public/clients/students/colleagues	Discussion with ACHD specialist indispensable
- Discuss workplace protective measures (face mask/PPE)	Early hospital admission/preferably at ACHD centre in case of clinical deterioration
**High-risk ACHD patients**	
Meticulous physical distancing	Consider early admission (even if asymptomatic)
Avoidance of direct contact to clients or students whenever possible. Preference for home office work	Discussion with ACHD specialist indispensable
	Early planning of treatment strategy in case of deterioration or intensive care therapy requirement

ACHD, adult congenital heart disease; Sars-CoV-2, Severe Acute Respiratory Syndrome Coronavirus 2.

Supplementary Figure 2. Medical management according to risk stratification [6]
Recommendations for work/education and general medical management by risk category.
The table is included in Reference 6.
